# miR-223: a key regulator of pulmonary inflammation

**DOI:** 10.3389/fmed.2023.1187557

**Published:** 2023-07-03

**Authors:** Mingyu Shi, Qianying Lu, Yanmei Zhao, Ziling Ding, Sifan Yu, Junfeng Li, Mengjun Ji, Haojun Fan, Shike Hou

**Affiliations:** ^1^Institute of Disaster and Emergency Medicine, Tianjin University, Tianjin, China; ^2^Wenzhou Safety (Emergency) Institute of Tianjin University, Wenzhou, China

**Keywords:** miR-223, inflammatory disease, acute lung injury (ALI), asthma, chronic obstructive pulmonary disease (COPD), COVID-19

## Abstract

Small noncoding RNAs, known as microRNAs (miRNAs), are vital for the regulation of diverse biological processes. miR-223, an evolutionarily conserved anti-inflammatory miRNA expressed in cells of the myeloid lineage, has been implicated in the regulation of monocyte–macrophage differentiation, proinflammatory responses, and the recruitment of neutrophils. The biological functions of this gene are regulated by its expression levels in cells or tissues. In this review, we first outline the regulatory role of miR-223 in granulocytes, macrophages, endothelial cells, epithelial cells and dendritic cells (DCs). Then, we summarize the possible role of miR-223 in chronic obstructive pulmonary disease (COPD), acute lung injury (ALI), coronavirus disease 2019 (COVID-19) and other pulmonary inflammatory diseases to better understand the molecular regulatory networks in pulmonary inflammatory diseases.

## 1. Introduction

The body’s non-specific immune response to injury or infection is called inflammation. It is a delicate matter of homeostasis and is highly controlled. Excessive inflammation can lead to organ dysfunction, tissue destruction or even malignancies. When injury or infection occurs, Toll-like receptors (TLRs), a family of pattern recognition receptors in sentinel cells can sense risk factors and then activate the signaling pathways associated with inflammatory responses. This results in the generation of chemokines and cytokines that possess inflammatory properties, thereby facilitating the attraction of other types of immune cells (1).

In mammals, the exchange of gases is facilitated by the lung, which serves as a crucial organ. The lung is in direct contact with the external environment and is the certain target of pathogens, allergens and poisons, which easily cause infection or inflammation, such as ALI, COPD and tuberculosis. In particular, the emergence of COVID-19 in December 2019, caused an unparalleled disturbance and drew a wide range of attention in human society (2). Thus, it is imperative to create secure and efficient therapies that can restrict inflammatory reactions in damaged lungs.

MicroRNAs (miRNAs or miRs) are a type of noncoding RNA molecules with a length of approximately 18–22 nt (3). They are highly conserved and single-stranded. miRNAs have the ability to control the expression of target genes at the posttranscriptional level (4). The targeting of a specific gene can be carried out by various miRNAs, and a particular miRNA can target several genes within a single cell type, which forms a complex regulatory network (5). Numerous studies have shown that miRNAs participate in several biological processes (6). In recent years, many studies have shown that miRNAs serve as biomarkers and treatments for different kinds of diseases (7), such as allergic diseases (8–11), cardiovascular disease (12–15), cancer (16–19) and diabetes (20–22).

The development and function of the myeloid lineage are dependent on miR-223, a microRNA that is conserved in vertebrates (23, 24). miR-223 is highly conserved in various organisms, its expression in myeloid lineage cells is characterized in humans and mice (25). miR-223 can be expressed in hematopoietic cells particularly in granulocytes and their precursors (26). During lineage commitment, miR-223 has been shown to inhibit the differentiation of progenitor cells into erythrocytes and facilitate their differentiation into granulocytes (27). It has been discovered by researchers that miR-223 is crucial in the regulation of inflammation in the lungs (28).

This review provides a comprehensive summary of the involvement of miR-223 in the inflammatory response across different cell types and its significance in pulmonary inflammatory diseases, aiming to enhance the understanding of clinical manifestations related to pulmonary inflammation.

## 2. The biogenesis, expression regulation and function of miR-223

**Figure 1:**
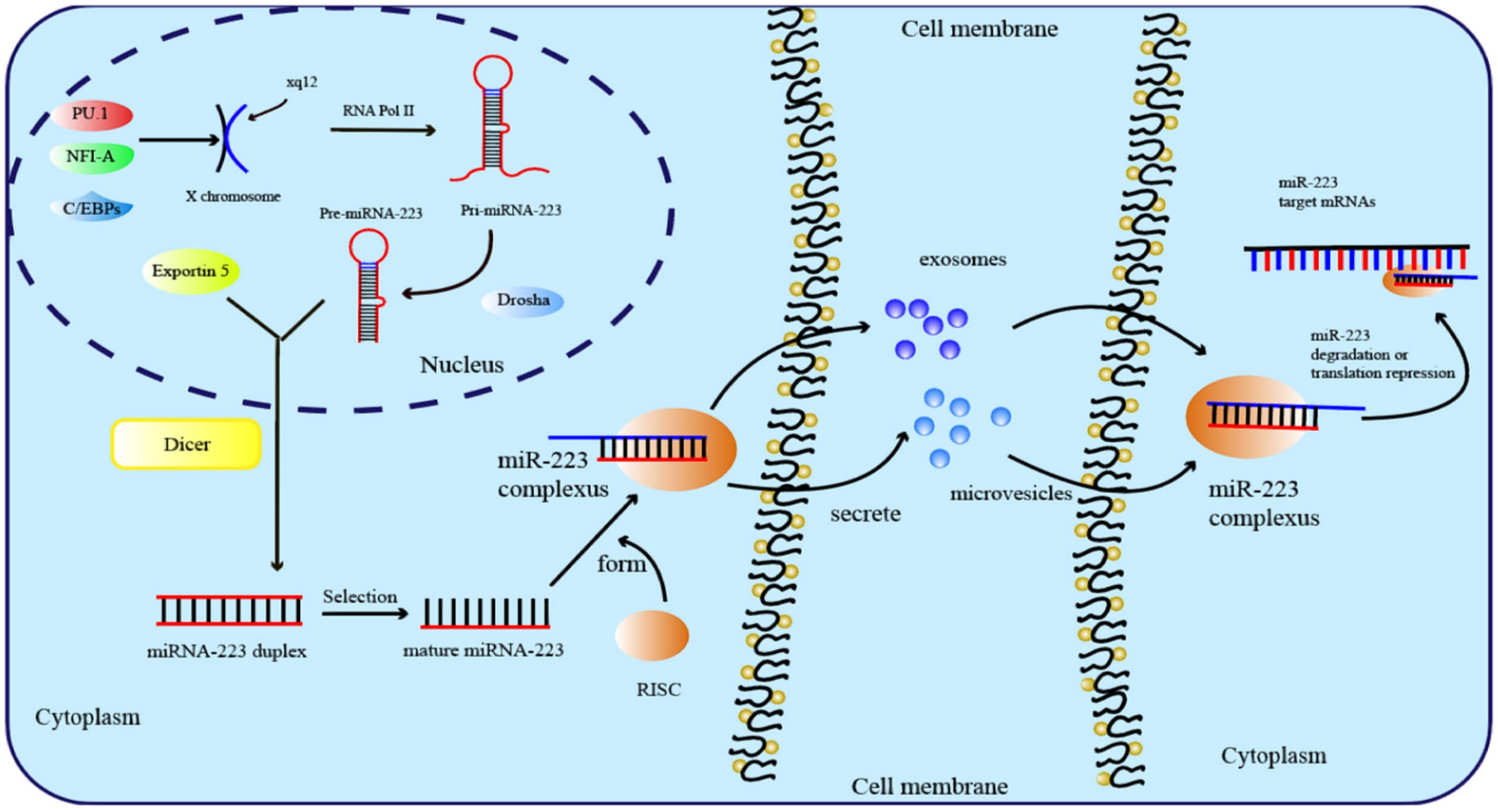
Overview of miRNA-223 biogenesis, function, and transfer.

During evolution, miR-223 has been highly conserved and is located at the q12 site of the X chromosome, with a length of 22 nt (29). Before mature miR-223 can bind to the complementary sequences in the 3′ or 5′ untranslated region, a complex biosynthesis process is necessary, as is the case with all miRNAs (30). The transcription of miRNA genes into primary transcripts (pri-miRNA) occurs through RNA polymerase II in the nucleus (31). The primary transcript of miR-223 is known as pri-miRNA-223, which contains a hairpin structure located in the third exon and primarily generates the miR-223-3p strand. Then, RNA endonucleases (RNase) catalyze pri-miRNA-223 via two successive cleavage steps (32). In the first step, the RNase III-type endonuclease DROSHA cleaves pri-miRNA-223 transcripts and releases a short oligo nucleotide known as pre-miRNA (33). After being recognized by Exportin-5, pre-miRNA-223 is exported to the cytoplasm for further processing by the RNase III enzyme Dicer (34). Finally, the RNA-induced silencing complex (RISC) is formally formed as mature miRNA-223 binds to the Argonaute protein (Figure 1) (35).

Several transcription factors control the expression of miR-223. Early in 2007, PU.1 and C/EBPs known as myeloid transcription factors, were found to drive the transcription of miR-223 (36). Furthermore, upstream of pre-miR-223, miR-223 expression can be upregulated directly by PPAR-γ regulatory elements in macrophages derived from bone marrow (37). In contrast, Krüppel-like Factor 6 (KLF6) is a negative regulator of miR-223, and the overexpression of KLF6 has been proven to downregulate the expression level of miR-223 in macrophages and promote the polarization of the M1 phenotype (38). The amount of miR-223 produced is also regulated by epigenetics. For example, Fazi et al. reported that in patients with acute myeloid leukemias, the recruitment of chromatin remodeling enzymes by AML1/ETO to the AML1 binding site on the pre-miR-223 gene results in the induction of heterochromatic silencing of miR-223 (39).

Functionally, miRNAs silence genes by causing mRNA destabilization, degradation, and translational repression through binding to the complementary 3′-untranslated regions (3′ UTRs) of mRNA (40). Long et al. demonstrated that miR-223 targets NLRP3 by directly binding to its 3′UTR, resulting in the inhibition of NLRP3 expression and subsequently suppressing inflammasome activation and pyroptosis in endothelial cells infected with *T. pallidum* (41). In neuroblastoma (NB), miR-223 can bind to the 3′UTR of FOXO1, resulting in decreased expression of FOXO1, thus increasing the malignant ability of NB cells (42). In breast cancer, miR-223 can directly bind to the 3′UTR of the tumor suppressor gene FBXW7 and consequently facilitate the infiltration and spread of breast cancer cells (43).

## 3. The role of miR-223 in the inflammatory response

During the inflammatory response, miR-223 expression undergoes changes in various cell types. Modulations the level of miR-223 expression can modulate the diverse functions of various cells, thereby ameliorating or aggravating the resulting tissue inflammation. During inflammatory responses, by binding to specific targets, miR-223 can regulate the differentiation and proliferation of granulocytes, macrophages, and DCs, as well as the polarization of macrophages. In addition, through binding to specific genes, miR-223 can suppress proinflammatory cytokines or inflammatory signals within these cells. This section aims to give a summary of how miR-223 contributes to various biological processes in granulocytes, endothelial cells, epithelial cells, macrophages and dendritic cells (Table 1).

**Table 1:** Targets and functions of miR-223 inside cells.

Cell	Target	Function	References
Granulocytes	Mef2c, NFIA, C/EBPα	Regulate granulocytes proliferation and differentiation	(44–46)
Macrophages	Pknox1, Rasa1, NFAT5, TRAF6, STATS, NLRP3	Regulate macrophage differentiation, polarization, and proinflammatory cytokine release	(38, 47–51)
Endothelial cells	ICAM-1, NLRP3	Attenuate endothelial cell inflammation	(52, 53)
Epithelial cells	NLRP3, microvesicles	Attenuate epithelial cell inflammation	(54–56)
Dendritic cells	NLRP3, C/EBPβ, Irak1, Rhob, Rasa1, Cfla, Kras	Regulate dendritic cell functions and influences immune-related protein networks	(57–60)

### 3.1. MiR-223’s involvement in the differentiation and activation of granulocytes

Granulocytes have three main granulocyte subsets: eosinophils, basophils and neutrophils. Within eosinophils, there exist sizable granules containing distinct eosinophilic proteins, such as eosinophil-derived neurotoxin, eosinophil peroxidase, major basic protein, and eosinophil cationic protein (61). The characteristic morphology of basophils is distinguished by the presence of large granules that are heavily stained and contain heparin, histamine sulfate, chondroitin, as well as other mediators like platelet-activating factor (PAF), slow-reacting kallikrein, neutrophil chemotactic factor (NCF). Under steady-state conditions, billions of neutrophils undergo apoptosis and are quickly replenished, as these cells have a short lifespan (62). During instances of inflammation or infections, emergency granulopoiesis is initiated, leading to a rise in the daily output of neutrophils (63). MiR-223 is highly expressed in the hematopoietic system and is a powerful regulator of granulopoiesis, with the highest expression in granulocytes. During the retinoic acid (RA)-induced granulocytic differentiation, inhibiting miR-223 expression reduces the efficiency of RA-induced differentiation of granulocyte-monocyte progenitors into monocytes (64, 65).

In 2008, Jonathan and his colleagues found that miR-223 can regulate granulocyte function and neutrophil progenitor cell proliferation by targeting myocyte enhancer Factor 2C (Mef2c) in mice. Hypermature and hypersensitive granulocytes, which display increased fungicidal activity upon activating stimuli, have been observed in mice lacking miR-223 (44). In the mouse model of allergic rhinitis (AR) inflammation, upregulation of miR-223 increased eosinophils granule protein expression, aggravated mucosal destruction and enhanced AR inflammation (66). When lung tissue injury occurs, miR-223 is rapidly upregulated in lung infiltrating granulocytes to attenuate injury during lung inflammation (67).

Studies have demonstrated that in the process of progenitor cell differentiation into granulocytes and monocytopoiesis, miR-223 is significantly upregulated by PU.1 and C/EBPβ known as myeloid transcription factors (45). Yang et al. utilized microarray profiling to assess the expression of miRNAs in neutrophils that were collected from patients suffering from severe traumatic injuries, they found that miR-223 participates in the traumatic pathogenesis of these patients (68). According to Vian’s research findings, the modulation of miR-223 activity has a significant influence on the differentiation and maturation of myeloid cell lines toward erythroid, granulocytic, monocytic, macrophagic lineages. Furthermore, overexpression of miR-223 promotes granulopoiesis and disrupts the differentiation of erythroid and monocytic macrophages (45). Other studies have also indicated that cytokines such as nuclear Factor I A (NFIA) can bind to the miR-223 gene promoter to repress its expression during the differentiation of granulocytes (46).

### 3.2. The function of miR-223 in regulating macrophage polarization

Macrophages are vital coordinators of immune activity and homeostasis. Based on temporal and environmental factors, macrophages can change their polarization direction to promote host immune defense mechanisms (69). In 2000, the nomenclature for macrophage polarization into M1 and M2 phenotypes was introduced (70). *In vitro*, cytokines have the ability to polarize macrophages. The classical activation of macrophages is initiated by LPS and IFN-γ, resulting in the development of a proinflammatory phenotype with pathogen-killing capabilities. These macrophages are commonly referred to M1 macrophages. Macrophages alternatively activated by IL-4 *in vitro* were named M2, which can promote cell proliferation and tissue repair (71). In the presence of pathological conditions, along with resident macrophages, additional macrophages are recruited to affected tissues and polarize into either M1 (proinflammatory) or M2 (anti-inflammatory) phenotypes.

Studies have shown that miR-223 can promote the polarization of macrophages toward the M1 and M2 phenotypes under different conditions. On the one hand, the downregulation of miR-223 can promote classic inflammatory M1-type macrophage activation. Chen’s study found that downregulation of miR-223, through its binding to signal transducer and activator of transcription 3 (STAT3), increases the production of proinflammatory factors like IL-6, and IL-1β (72). Further research revealed that in macrophages, by targeting Ras homolog gene family member B (Rhob), the downregulation of miR-223 can enhance NF-κB signaling pathways and the mitogen-activated protein kinase (MAPK). This, in turn, induces the polarization of M1 proinflammatory macrophages and increases production of proinflammatory cytokines (73).

On the other hand, the upregulation of miR-223 can promote M2 (anti-inflammatory) phenotypes. Zhuang et al. discovered that in diet-induced adipose tissue inflammation, miR-223 can trigger macrophages to differentiate into an anti-inflammatory M2 phenotype to alleviate adipose tissue inflammation caused by a high-fat diet (74). Also, miR-223 expression was found to be significantly downregulated in heart tissues and heart-infiltrating macrophages in a mouse model of coxsackievirus B3 (CVB-3)-induced viral myocarditis. miR-223 directly targeting and inhibiting the expression of Pknox 1, thus to suppressed the expression of the M1 marker level and promoted the M2 marker level (47). Similarly, He and his colleagues found that miR-223 carried by exosomes can be taken in by macrophages and induce them to differentiate into M2 phenotype to accelerate wound healing by controlling Pknox1 gene expression. These studies revealed the role of the miR-223-Pknox1 axis in macrophage polarization (48). Wang et al. showed that miR-223 can mitigate sepsis by binding to the mRNA of NFAT5 and Rasa1, leading to IL-4-mediated differentiation of M2 macrophages (49). Moreover, a study on viral myocarditis (VMC) in mice discovered that long noncoding RNA maternally expressed gene 3 can facilitate miR-223 expression in macrophages, inhibiting M1 and promoting M2 macrophage polarization. Thus, upregulated miR-223 can inhibit TNF receptor-associated Factor 6 (TRAF 6) and suppress myocarditis and inflammation via NF-κB pathway inactivation in VMC mice (50). Additionally, Wang and his colleagues found that in macrophages, overexpression of miR-223 can reduce NF-κB activation by inhibiting IL-1 receptor-associated kinase-1 (IRAK-1), which leads to the polarization of M2 macrophages (75). In summary, at the molecular level, by targeting Pknox1, Rasa1, NFAT5, and TRAF6, upregulated miR-223 can induce polarization of macrophages toward the M2 anti-inflammatory phenotype during inflammation.

To summarize, miR-223 plays a crucial role in regulating the balance between M1 or M2 macrophage polarization and the development of inflammatory diseases.

### 3.3. The function of miR-223 in regulating inflammation in endothelial cells

The endothelium plays a critical role in maintaining multiorgan health and homeostasis. The healthy endothelium has a variety of biological functions, such as acting as a semi-permeable barrier, regulating the exchange and transport of substances, maintaining innate immunity, and balancing the production of vasodilators to regulate vascular tone, accelerating re-endothelialization to repair vascular injury and secreting antiplatelet and anticoagulant molecules to regulate hemostasis and regulating angiogenesis by producing factors (76). Lung endothelium undergoes continuous stretching during respiration and remains consistently exposed to external environmental substances. Impairment of the lung endothelium has been observed in various clinical conditions, such as acute respiratory distress syndrome (ARDS), COPD, pulmonary fibrosis, pneumonia, autoimmune disorders, pulmonary hypertension, and additional ailments (77). miR-223 plays an important role in endothelial cell inflammation and vascular endothelial injury.

On the one hand, miR-223 regulates endothelial cell inflammation. Upregulated miR-223 can be observed in peripheral microvesicles (MVs) in the plasma samples of nephritis, enteritis, hepatitis and atherosclerosis patients. miR-223 derived from platelets is conveyed to human umbilical vein endothelial cells (HUVECs) through peripheral MVs (78). Li and colleagues demonstrated that the transfection of miR-223 into HUVECs under TNF-α stimulation resulted in the inhibition of intercellular adhesion molecule-1 (ICAM-1) level. These findings suggest that miR-223 is a crucial factor in platelet-derived exosomes and is involved in inflammation response by impeding the phosphorylation of p38, JNK, and ERK, and hindering the nuclear translocation of NF-κB p65 (52). Another investigation demonstrated that platelets released microparticles (PMPs) containing functional miR-223 during sepsis can reduce the expression of ICAM-1 in endothelial cells, thereby potentially providing protection against excessive vascular inflammation induced by sepsis (79). In addition to the NF-κB and MAPK signaling pathways, the NLRP3 inflammasome pathway can also be regarded as a potential therapeutic target in vascular inflammation. The inhibition of NLRP3 inflammasome in endothelial cells through tree peony bark (Pae) leads to an increase in the expression of miR-223 in exosomes derived from the plasma of hyperlipidemic rats (53). What is more, Pae-exo increased the expression of miR-223 and decreased the expression of NLRP3, apoptosis-related spot-like protein (ASC), caspase-1 and ICAM-1 to relieve the endothelial dysfunction in atherosclerosis (AS) (80). Moreover, Zhang and his colleagues’ study on aortic damage caused by Se deficiency revealed that miR-223 is significantly downregulated, which leads to an increase in the expression of NLRP3 and the downstream targets, such as ASC, caspase-1, IL-18 and IL-1β (81).

On the other hand, miR-223 is a crucial regulator of vascular endothelial injury. Wang noted that miR-223 can reduce vascular endothelial injury by inhibiting the production of IL-6 and TNF-α during Kawasaki disease (KD) (82). Moreover, differential miR-223 expression in vascular endothelial cell (VEC) extracellular vesicles (EVs) can influence VEC generation and apoptosis. MiR-223-3p overexpression can reduce injury to mouse cardiac microvascular endothelial cells (MCMECs) and inhibit endothelial cell apoptosis in mice by regulating the expression of NLRP3 (83). In addition, Alexandre demonstrated for the first time that activation of neutrophils with various inflammatory stimuli induces the release of EVs that are internalized by endothelial cells, thus leading to the transfer of miR-223 and subsequent endothelial damage (84). These studies show that miR-223 can attenuate endothelial cell injuries.

### 3.4. Role of miR-223 in epithelial inflammation

Epithelial tissues constitute the physical barrier between our internal tissues, organs, etc. and the external environment exposes individuals to a wide range of inflammatory stimuli. The barrier is established through the action of stem cells that maintain a balance between self-renewal and differentiation to replenish lost cells and facilitate tissue repair in response to injury (85). Epithelial cells possess pattern recognition receptors that allow them to detect and react to various signals. These signals can guide the response of epithelial stem cells through signaling pathways that strengthen barrier function, signal nearby epithelial cells, attract immune cells, and promote tissue repair (86). The lung is a highly intricate organ consisting of different sections, which are covered by numerous specialized epithelial cells that have evolved to facilitate the exchange of gases necessary for the survival of air-breathing organisms on land. Under normal conditions, the epithelial cells in the lungs of adult mammals exhibit remarkable quiescence. Acute respiratory distress syndrome, coronavirus-induced acute lung injury, childhood interstitial lung diseases, cystic fibrosis, chronic obstructive pulmonary disease, and pulmonary fibrosis syndromes all involve, to some extent, dysfunction of the lung epithelium as a contributing factor (87).

miR-223 plays crucial functions in epithelial cells. Besides controlling the process of growth and morphology (88), the analysis of bioinformatic has shown that NLRP3 is a vital target of miR-223 in regulating epithelial cells. In renal tissue injury of mouse model induced by LPS, upregulated miR-223 can inhibit TXNIP and the NLRP3 inflammasome and attenuate LPS-induced injury in proximal tubule epithelial cells (54). In lung epithelial cells, upregulation of miR-223 by STIM1 can alleviate influenza A virus induced inflammatory injury in lung epithelial cells by deactivating NLRP3 and inflammasomes (55). Moreover, suppression of miR-223 in A549 cells induced by B(a)P resulted in elevated levels of TNF-α and IL-6 in the supernatant, as well as increased protein level of NLRP3, IL-1β, IL-18, caspase-1 (56). A recent study from Ren revealed that in animal models of dry eye (DE), miR-223 can suppress inflammation induced by hyperosmolarity in corneal epithelial cells by reducing the activation of NLRP3. The fact that miR-223 expression levels are inversely correlated with NLRP3 expression levels indicates that selectively increasing miR-223 expression could be a potential approach to alleviate chronic inflammation in DE (89). In addition to targeting NLRP3, in a model of pulmonary arterial hypertension (PAH), miR-223 can suppress the expression of the Integrin-β 3 subunit gene (ITGB3) to alleviate the progression of PAH and avoid the dysfunction of pulmonary arterial endothelial cells (90). What is more, by targeting to Rhob and deactivation of NF-κB gene activity in dairy cows, miR-223 attenuated LPS-induced inflammatory responses in mammary epithelial cells (91). Thus, through diverse signaling pathways, miR-223 reduces tissue damage by regulating the epithelial inflammation process.

### 3.5. The function of miR-223 in the differentiation of dendritic cells

DCs, which are antigen-presenting cells, have vital role in maintaining and inducing immunity as well as tolerance (92). DCs are bone marrow-derived cells arising from lympho-myeloid hematopoiesis that coordinate innate and adaptive immune responses (93). DCs exist in two functional states: immature and mature. Recognition of tissue homeostasis disturbances through damage-associated molecular patterns or pathogen-associated molecular patterns leads to DC maturation (94). DCs are critical innate immune cells at barrier sites, including the lung, playing a decisive role in initiation of adaptive immune responses against foreign material, infection, commensals or tissue damage (95). DCs are present in various regions of the lung tissue, positioned beneath the epithelial layer, ready to come into contact with foreign substances, infections, or tissue damage. In this capacity, they benefit from their capability to actively collect samples from the airways (96).

In process of hematopoietic stem cell (HSC) differentiation into DCs, the miR-223 expression level is altered in myeloid stem cells, HSCs and DCs, which proves that miR-223 takes part in DC differentiation (97). Bros et al. discovered that miR-223 has a negative regulatory effect on DCs activation in response to inflammatory. Upon DCs being stimulated with LPS, a significant decrease in miR-223 expression was observed. In response to glucocorticoids and anti-inflammatory cytokines, miR-223 expression is increased in DCs during the differentiation of bone marrow cells into BMDCs. Subsequently, the high level of miR-223 contributes to decreased Cfla, Kras and Rasa1 mRNA expression and influences immune protein regulatory networks (57). Tang’s research revealed that miR-223 binding to Rhob can also regulate DC differentiation and inhibit antigen uptake and presentation (98). In the autoimmune myocarditis (EAM) mouse model, Chen observed that miR-223 expression is markedly reduced in comparison to normal mice, whereas overexpressing miR-223 in DCs suppresses NLRP3 inflammasome production and facilitates the polarization of DCs into a tolerogenic phenotype (58). In mouse model of allogeneic heterotopic heart transplantation, overexpression of miR-223 in immature dendritic cells was found to result in longer graft survival and reduced infiltration of immune cells. The regulation of DC function by miR-223 through Irak1, Treg differentiation, and IL-10 secretion suppressed allogeneic heart graft rejection (59). In addition to EAM, miR-223 has been shown to regulate function and differentiation of DCs by targeting C/EBP-β in a mouse model of colitis. When miR-223 was deficient, monocytes produced more proinflammatory cytokines and gave rise to more monocyte-derived DCs upon stimulation (60). In conclusion, the aforementioned research demonstrates that miR-223 operates as a repressor of DC activation and sustains a state of maturation resistance that promotes a tolerogenic response during instances of inflammation.

## 4. The role of miR-223 in pulmonary inflammation

The alveoli constitute a delicate tissue structure, which makes the regulation of pulmonary inflammation as important as that of the respiratory tract. The extensive surface area of the lung is crucial for its vital role in gas exchange, and it also act as the primary barrier against the external environment. Due to its constant exposure to an airflow containing various types of proinflammatory stimuli, such as radioactive, infective, pro-allergenic or toxic agents, the lung plays a significant role in protecting the body from harmful environmental factors (99). Although the inflammatory response in the lung is thought to be a straightforward series of reactions, inflammation is a delicate matter of homeostasis and is tightly controlled. Excessive inflammation will lead to acute organ dysfunction and multiorgan failure or chronic tissue remodeling and destruction.

**Figure 2:**
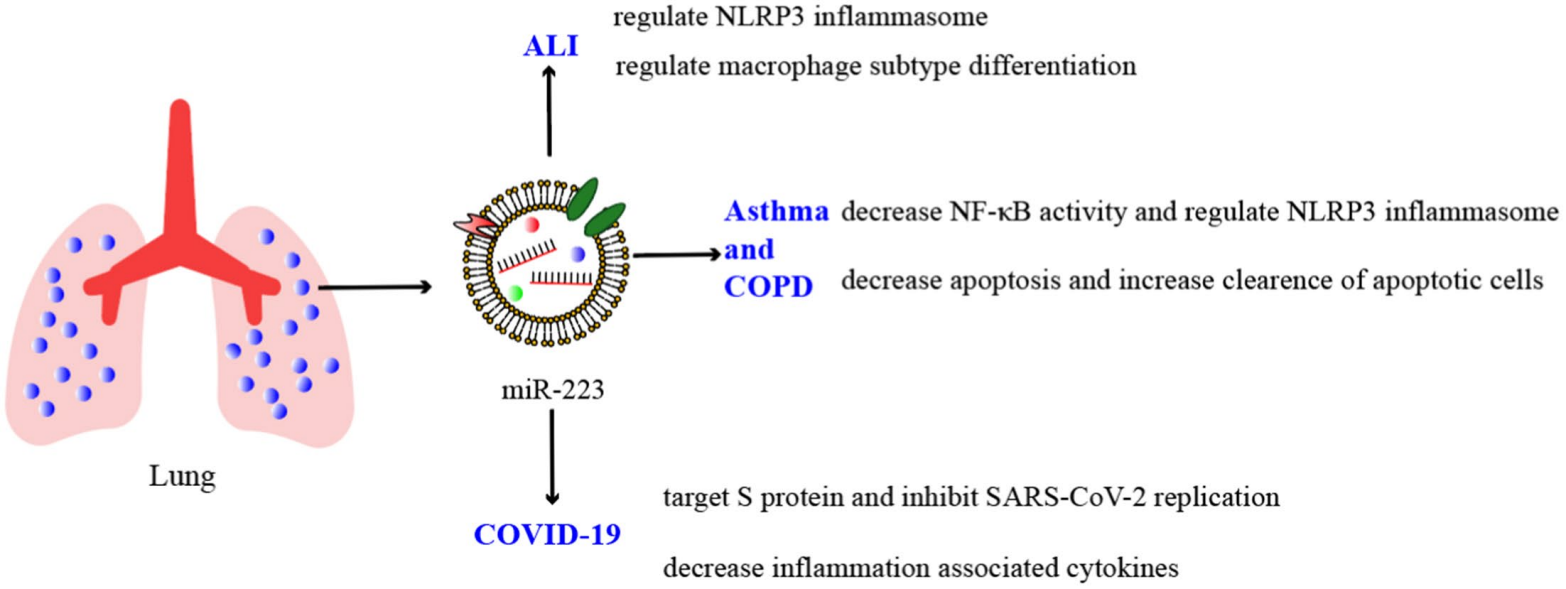
Schematic illustration of miR-223’s mode of action in pulmonary inflammation.

As we mentioned before, miRNAs are crucial regulators of various developmental and cellular processes. In certain pulmonary inflammatory conditions like pneumonia, COPD, and tuberculosis, miR-223 can target specific genes and suppress the synthesis of inflammatory mediators or hinder inflammation signaling pathways, thereby safeguarding the body from inflammatory damages (Figure 2; Table 2).

**Table 2:** miR-223 expression varies across different cell types and is influenced by various conditions.

Expression of miR-223	Cell lines/patients samples	Condition	References
Overexpression of miR-223	Porcine lung	↓ NLRP3 activity	(100)
Overexpression of miR-223	Bronchial epithelial cells	↓ NF-κB activity	(101)
Down-regulation of miR-223	A549 cells	↑ Rhob, NLRP3, TLR4/NF-κB activity	(73)
Overexpression of miR-223	Macrophages derived from BMDMs	↓ TLR4/NF-κB activity	(102)
Down-regulation of miR-223	HBECs	↑ NF-κB activity	(101)
Overexpression of miR-223	HBECs	↓ PARP-1 activity in blood	(103)
Down-regulation of miR-223	PBMCs	↑ IKKα activity	(104)
Overexpression of miR-223	A549 Cells	↓ NLRP3 activity in PBMCs	(105)
Overexpression of miR-223	Neutrophils/airway smooth muscle cells/Lewis lung carcinoma cells	↓ IGF-1R activity	(106)
Down-regulation of miR-223	A549 cells/Lung cancer cells	↓ TGF-β receptor 3	(107)
Overexpression of miR-223	Lewis lung carcinoma cells	↓ CDK2 activity	(108)
Down-regulation of miR-223	glioblastoma	↓ IL-1β, IL-8, IL18, MCP-1 activity	(109)
Down-regulation of miR-223	adipose stem cells	↓ IL-6, IL-1β and TNF-α activity	(110)

### 4.1. Acute lung injury

ALI along with its severe form named ARDS, are both acute inflammatory lung conditions that can result in significant morbidity and mortality rates every year (111). ALI/ARDS can result from a range of lung injuries, including direct injuries like embolism, inhalation of toxic gases, lung contusion, and pneumonia, as well as indirect injuries such as major trauma, hemorrhagic shock, sepsis, drug overdose, or reperfusion injury (112, 113). Direct and indirect lung injuries exhibit comparable final pathophysiological features, such as compromised alveolar-capillary membrane function, reduced alveolar fluid clearance, and increased inflammation, which can lead to gas exchange difficulties and hypoxemia (114, 115). Although there have been extensive studies on ALI/ARDS, there are still no specific medical treatments. Therefore, the study of specific treatment strategies is urgently needed.

**Figure 3:**
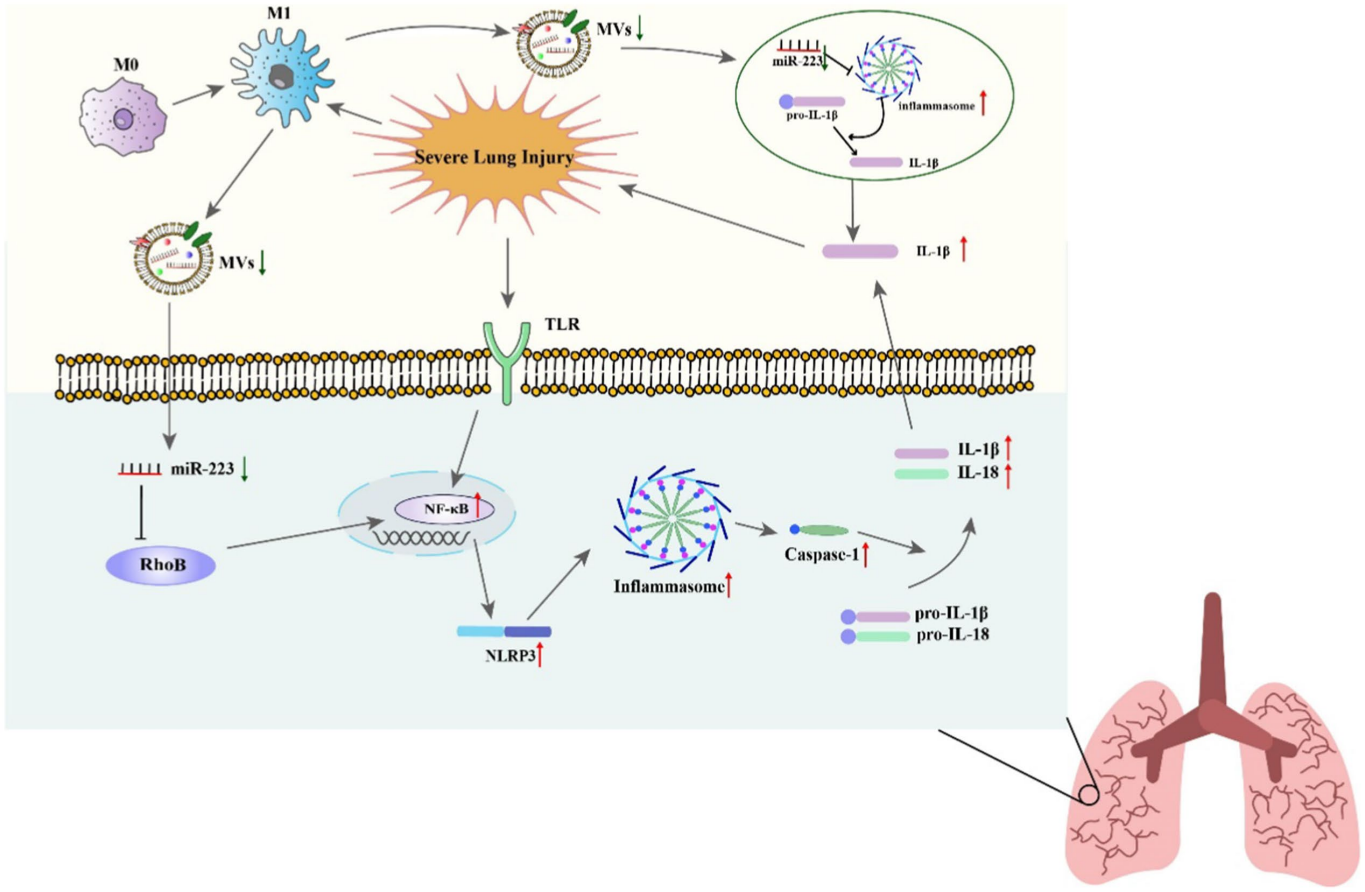
The roles of miR-223 in regulating acute lung injury mechanisms.

Studies have indicated that miR-223 can regulate the inflammatory response during ALI/ARDS (116). Reduced miR-223 expression has been demonstrated in *in vitro* experiments to diminish the NLRP3 inflammasome, the inhibition of RHOB and TLR4/NF-κB signaling pathway, leading to the exacerbation of lung injury (73). A further study found that miR-223 overexpression can alleviate LPS-induced ALI/ARDS *in vivo* by directly targeting NLRP3 (117, 118). Moreover, in macrophages, miR-223 can regulate subtype differentiation (73). miR-223 has been found to regulate macrophage differentiation by targeting the NLRP3 inflammasome, as shown in studies using a mouse model where lung inflammation is mediated by macrophages (28). In septic mice, miR-223 is an important regulator of the M2-type polarized target genes Nfat5 and Rasa1 mediated by IL-4 (49). What is more, treat with miR-223 from epithelium and endothelium derived exosomes can regulate the immune balance of alveolar macrophages (AMs) by targeting RGS1 mediated calcium signaling-dependent immune response (119). Furthermore, He’s study showed that miR-223 can also alleviates ALI by targeting STK39 in AMs (120). These results indicate that miR-223 can regulate the immune balance of AMs and can be used as potential therapeutic drugs for ALI/ARDS (Figure 3).

miR-223 is also an inhibitor of neutrophil development and function, and it can alleviate neutrophilic airway inflammation by inhibiting the release of IL-1β and the NLRP3 inflammasome. miR-223 can restrict the differentiation of Ly6G+ neutrophils derived from bone marrow and suppress the activity of the NLRP3 inflammasome and the production of IL-1β in ALI caused by mitochondrial damage-associated molecular patterns. However, the lack of miR-223 leads to continuous activation of NLRP3 and IL-1β (100). Moreover, neutrophil-derived microRNAs can modify mucosal gene expression during acute lung injury by transferring to pulmonary epithelial cells. In mouse neutrophils, miR-223 can be transferred to lung epithelial cells through MVs, where it inhibits poly (ADP-ribose) polymerase 1 (PARP-1) to impede acute lung injury. Therefore, overexpression of miR-223 in mice provides protection during acute lung injury (121). Additionally, Tan proved that miR-223 directly targeted high-mobility group box 2 (HMGB2) gene and the downregulation of miR-223 increased HMGB2 protein level, which activated the JNK signaling pathway and thus induced oxidative stress and autophagy in LPS-treated alveolar epithelial cells. Knockdown of HMGB2 protein deactivated the JNK signaling pathway and inhibited autophagy and oxidative stress in alveolar epithelial cells (122). In summary, miR-223 is regarded as a potential therapeutic target in ALI as it can regulate the process of inflammation in ALI.

### 4.2. Asthma and COPD

COPD and asthma represent the most frequently occurring chronic inflammatory respiratory conditions (123, 124). Asthma is a frequent inflammatory condition affecting the lower respiratory tract and is characterized by airway obstruction and hyperresponsiveness (124, 125) with symptoms such as wheezing, difficulty breathing, chest tightness, and coughing (126). COPD is a diverse disease marked by lung inflammation, rapid decline in lung function, chronic bronchitis, and damage to the alveolar tissue caused by pulmonary inflammation (124, 127). This persistent inflammation results in symptoms such as breathlessness, chronic cough, and wheezing (128). Asthma and COPD are among the diseases with the highest incidence and socioeconomic burden worldwide. Therefore, research on asthma and COPD is extremely urgent.

Studies have shown that miR-223 is involved in the processes of COPD and asthma. miRNA profiling shows that compared to healthy groups, miR-223 is more highly expressed in bronchial brushings (129) and induced sputum supernatant, the stimulated secretion of fluid from the respiratory tract containing various inflammatory cells, which quantitative cell count is the reference standard to reflect the airway inflammation of asthma patients (130, 131). Likewise, in neutrophilic asthma patients, the expression level of miR-223 is increased compared to that in healthy controls and eosinophilic asthmatic patients (131). Ezzie et al. analyzed the differential expression of miRNAs in lung tissues of smokers with or without COPD and found that miR-223 expression levels were higher in patients with COPD. A recent study showed that miR-223 had good combinatory predictive ability in differentiating between health and mild COPD. Furthermore, miR-223 was correlated with airway eosinophilia and were able to distinguish pure eosinophilic COPD from other airway inflammatory subtypes (132). In addition, Hirai found that miR-223 had lower expression levels in asthma-COPD overlap (ACO) patients and could discriminate between ACO patients and patients with either asthma or COPD (133). These findings highlight that miR-223 expression is distinctively regulated in obstructive lung diseases (134).

**Figure 4:**
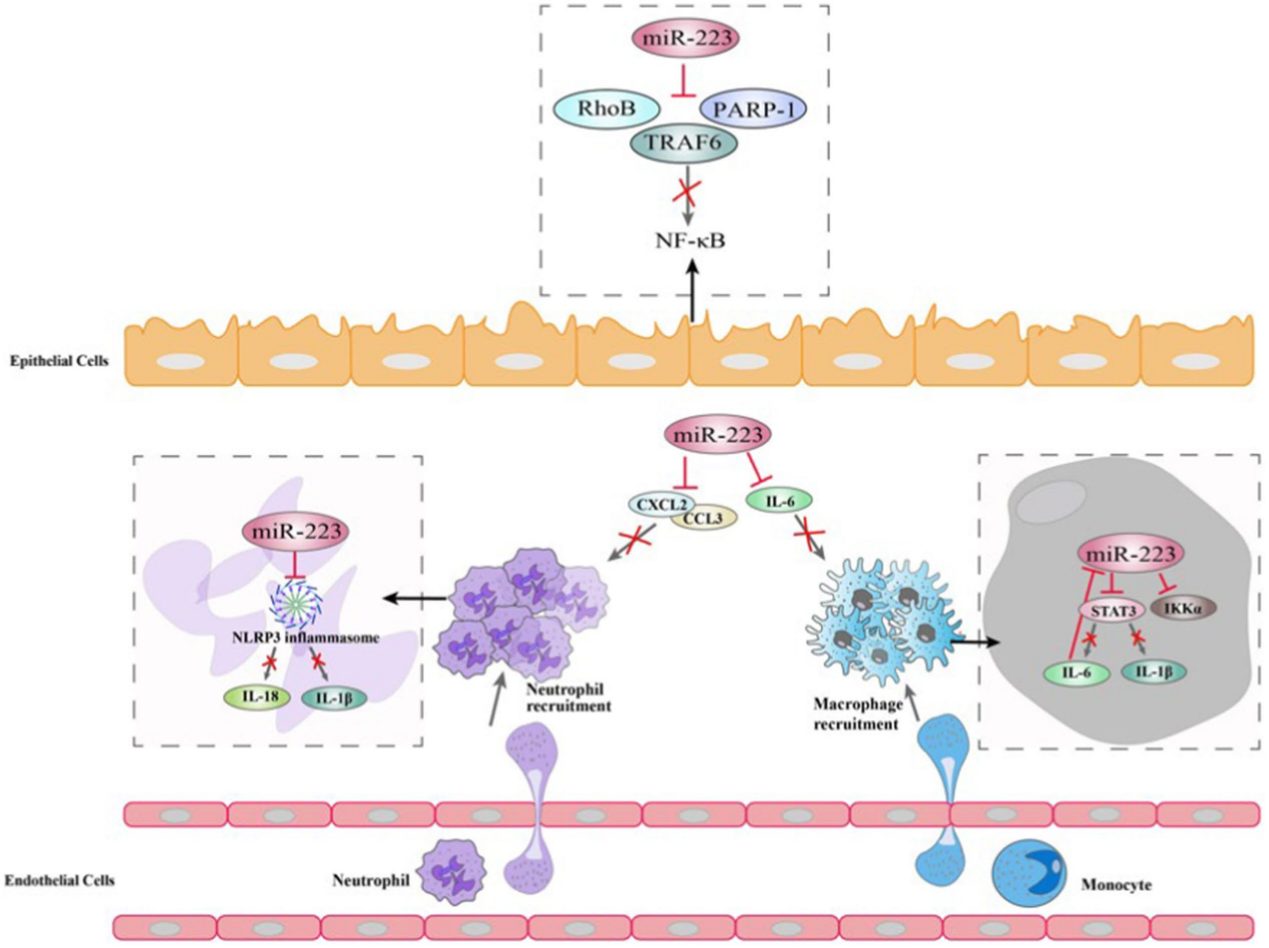
Mechanisms of miR-223 in the modulation of Chronic obstructive pulmonary disease.

The NF-κB signaling pathway can be triggered by environmental stimuli in the bronchial biopsies of patients with COPD and asthma, resulting in proinflammatory reactions (101). Several studies suggest that elevated levels of miR-223 in human bronchial epithelial cells due to overexpression can decrease NF-κB activity by influencing the activation of NF-κB targets such as PARP-1 (103) and IκB kinase α (IKKα) (135). On the one hand, PARP-1 activation is observed *in vitro* in response to cigarette smoke and oxidative stress (136). miR-223 can repress PARP-1 to reverse the excessive inflammatory response (121). On the other hand, during human monocytes differentiate to macrophages, the increase in IKKα levels is correlated with a decrease in miR-223 expression (135). The levels of IKKα in peripheral blood mononuclear cells were found to be similar between asthma and COPD patients and healthy groups, according to some researchers. However, COPD patients and control smokers have higher p-IKKα levels than nonsmoking controls (104). A further study revealed that miR-223 can regulate the expression of mucin 5 AC (MUC5AC), eotaxin-2 (CCL24) and thymic stromal lymphopoietin (TSLP) by targeting NF-kB signaling pathway, this suggests that miR-223 is a regulator of allergic inflammation and could potentially consider as novel and targeted therapy for asthma (117) (Figure 4).

In addition to the inflammatory response, important characteristics of asthma and COPD also include cell dysfunction and death (137). Insulin-like growth factor-1 receptor (IGF-1R), known as the controller of cell proliferation, has been confirmed as a target of miR-223. Liu’s research confirmed that miR-223 targets the 3′UTR of IGF-1R, which subsequently led to the suppression of IGF-1R expression (106). IGF-1 can activate PI3K/Akt and mTOR to control apoptosis, survival, growth, and cell proliferation by binding with IGF-1R (138). Abnormal IGF-1 signaling has been found in asthma and COPD (139). Moreover, a reduction in serum IGF-1 levels has been observed in COPD patients experiencing acute exacerbation in comparison to healthy individuals and stable COPD patients. Conversely, IGF-1 mRNA levels in lung tissue are elevated in COPD patients when compared to healthy people (140). In addition to IGF-1R, miR-223 also targets Mef2c, which is a transcription factor that promotes the proliferation of myeloid progenitors to inhibit the proliferation of myeloid progenitor cells. Within the bronchial epithelial cells of individuals with severe asthma, the expression of Mef2c is decreased compared to that in healthy controls (141).

In addition to cell differentiation and proliferation, several vitro studies have explored the impact of miR-223 on cell survival, invasion, and apoptosis. The contribution of TGF-β and its receptors to airway remodeling in COPD and asthma is well-established, and miR-223 can target TGF-β receptor 3 to promote cell invasion and viability and reduce apoptosis (142). In the serum of COPD patients and in bronchial biopsies of asthma patients, the expression of TGF-β1 is increased (143), while the expression levels of TGF-β1 observed in both bronchial epithelial cells and alveolar macrophages of COPD patients are decreased compared to control groups (107). This may potentially be attributed to elevated expression levels of miR-223 in individuals suffering from asthma and COPD. Other research has also revealed that miR-223 participates in cell death by targeting p53 and cyclin-dependent kinase 2 (CDK2) to inhibit migration and proliferation (108). While p53 expression is typically low during homeostasis, it can be induced by oxidative stress in airway epithelial cells, such as those exposed to smoke, leading to cell apoptosis (144). Although miR-223 levels are increased in COPD patients, its validated target p53 expression levels are found to be higher in the lung tissue of COPD patients as compared to non-COPD controls, particularly in smokers with COPD (145). This indicates that miR-223 may not effectively reduce apoptosis by regulating p53 in COPD patients compared to non-COPD people. Moreover, MO’s study showed that the expression levels of miR-223 was reduced and the expression level of NLRP3 was increased by lncRNA GAS5, which subsequently promoted pyroptosis in COPD (146). In addition, Xu and his colleges found that overexpression of miR-223 via treatment with miR-223 agomirs attenuated airway inflammation, NLRP3 levels and IL-1β release. This revealed a crucial role for miR-223 in regulating the immune inflammatory responses by depressing the NLRP3/IL-1β axis in neutrophilic asthma (147).

In summary, miR-223 can target several genes, such as CDK2, TGF-β, IGF-1R, Mef2c and p53, to regulate cell viability, cell invasion and cell apoptosis during asthma and COPD.

### 4.3. COVID-19

An uncontrolled or excessive innate immune response in individuals with COVID-19 can result in the development of ARDS and a cytokine storm, which manifests as severe alveolar inflammation. Usually, COVID-19 causes mild respiratory symptoms, but it can also lead to severe complications. When COVID-19 infects macrophages, they present viral antigens to T cells, which produce cytokines associated with different kind of T-cell subtypes. The subsequent release of large quantities of cytokines amplifies the immune response, resulting in a cytokine storm. Massive secretion of chemokines and cytokines like TNF-β, MCP-1, IL-8, IL-21, IL-6, and IL-1 is promoted in cells in response to COVID-19 infection. The cytokines and chemokines then recruit leukocytes and lymphocytes to infection site. CD8 T cells are able to generate potent mediators that can effectively eliminate COVID-19, but the ongoing release of these mediators may result in viral persistence and have a detrimental impact on the activation of CD8 T cells (148).

When COVID-19 infects a host cell, its S protein binds to the dipeptidyl peptidase-4 receptor (DPP4R) and causes genomic RNA to appear in the host cell cytoplasm. TLR3 is then sensitized by dsRNA, activating the signaling cascades of nuclear factor-κB (NF-κB) and interferon-regulatory factors (IRFs), which produce interferons (IFNs) and proinflammatory cytokines. IFN-III, IFN-II, and IFN-I are induced by engagement of IFN receptors, which also activate different members of the signal transducer and activator of transcription families and Janus kinase (JAK), forming specific transcription factor complexes. At the same time, JAK1 and JAK2 are activated by IFN-II, resulting in the formation of a phosphorylated STAT1 homodimer (149).

Active peptide angiotensin II (Ang II), converted from the inactive decapeptide Ang I from the renin-angiotensin-system (RAS), can lead to chronic tissue injury, hypertension and vasoconstriction through the JAK–STAT signaling pathway. Furthermore, the RAS mediates the production of proinflammatory cytokines. Ang II increases the infiltration of immune cells, which leads to the local production of proinflammatory cytokines such as IL-1, IL-6, IFN-γ and TNF-α in target tissues (150). Moreover, some studies have shown that macrophage infiltration along with the apoptosis of epithelial cells and pneumocytes occurs in lung tissue (151). This may be mediated by MCP-1 through TGF-β and TNF-α, which leads to cytokine production.

Some miRNAs have been reported to be vital for virus entry into host cells and ACE2 level regulation (152). Furthermore, diverse studies have demonstrated that miRNAs suppress the replication and expression of the SARS-CoV-2 spike protein (153). Considering this, miRNAs can play a positive or negative role during virus-related processes in different ways: binding to host transcripts; binding to viral transcripts and direct binding to the viral genome (154). In humans, miRNAs have the ability to serve two functions, which include either enhancing the durability and propagation of viral RNA or strengthening the host’s anti-viral response. Due to their potential to regulate these responses, miRNAs are regarded as potential instruments for exploring the networks that regulate the immune response to both COVID-19 infection and vaccination (155).

Wang et al. conducted a study and showed that miR-223-3p could directly target the S protein and inhibit the replication of SARS-CoV-2 (153). Which indicate that there is a correlation between low levels of circulating miR-223 and increased SARS-CoV-2 replication in elderly individuals and those with diabetes. Patients who are older and have comorbidities are at an increased risk of developing severe complications and experiencing higher mortality rates from COVID-19 as a result of infection with SARS-CoV-2.

**Figure 5:**
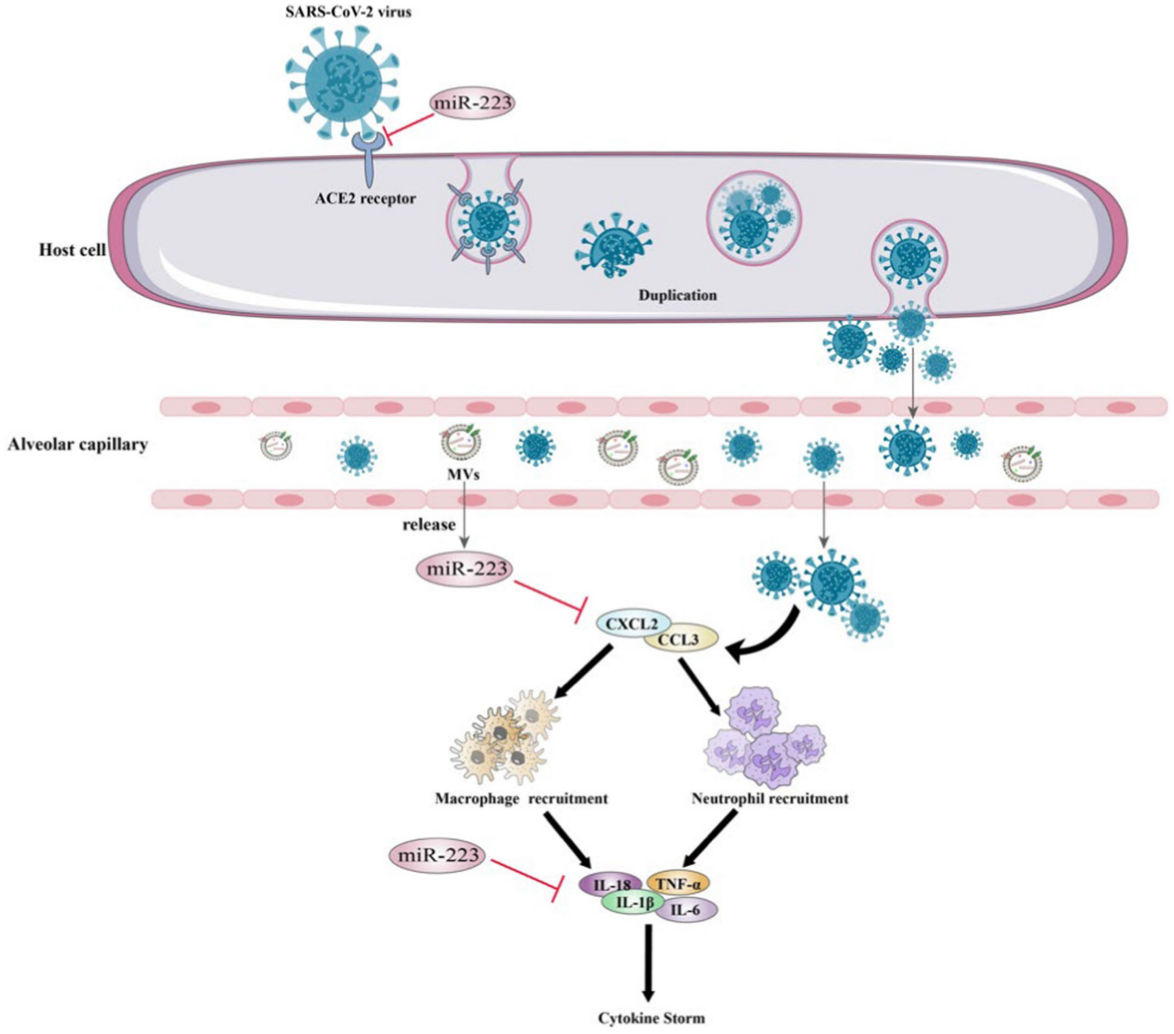
Mechanisms of miR-223 in the modulation of COVID-19.

In addition to targeting the S protein to inhibit SARS-CoV-2 replication, miR-223 may play a crucial role in cytokine storm regulation. During the COVID-19 pandemic, several studies have suggested a correlation between the cytokine storm in patients’ bodies and severe deterioration of their health (156). Ding et al. conducted an *in vitro* study on glioblastoma and found that treated with miR-223-3p mimic reduced the expression of several inflammation-associated cytokines, including IL-8, IL-18, IL-1β and MCP-1 resulting in inhibited cell proliferation and migration (109). Moreover, it has been demonstrated that miR-223-3p directly impacts the expression of inflammatory cytokines in adipose stem cells, which are known to be associated with cytokine storms (110) (Figure 5). These researches suggest that miR-223 can regulate cytokine levels in COVID-19 and thereby regulate the inflammatory process and disrupt the immune response.

### 4.4. Other lung inflammatory diseases

In addition to the diseases mentioned above, miR-223 also participates in the inflammatory response of other lung inflammatory diseases, including pulmonary tuberculosis (TB), sarcoidosis and pulmonary fibrosis (Table 3).

**Table 3:** The role of miR-223 in other lung inflammatory diseases.

Expression of miR-223	Cell lines/patients samples	Condition	References
Upregulation of miR-223	Lung tissue in tuberculosis mice	↓ CXCL2, CCL3 and IL-6 to delete Cxcr2	(157)
Upregulation of miR-223	The mouse macrophage J774A.1 cell line infected with *Mycobacterium tuberculosis* H37Rv	miR-223 modulates IKKα expression thus ↓ NF-κB activation	(158)
Downregulation of miR-223	Patients with pulmonary TB	↑ STAT1 and affect the interferon signaling pathway	(159)
Downregulation of miR-223	Peripheral blood and bronchoalveolar of sarcoidosis patients	↑ NF-κB apoptosis and proliferation signals	(160)
Downregulation of miR-223	Sarcoid patients	↑ NLRP3 activity	(161)
Upregulation of miR-223	Sputum supernatants in children infected with Aspergillus	Response to infection and enhanced airway inflammation induced by Aspergillus	(162)
Downregulation of miR-223	Rats with pulmonary fibrosis	↑ Hydroxyproline activation	(163)

TB, as a typical infectious disease, is a chronic illness distinguished by continuous inflammation (164, 165). TB is caused by *Mycobacterium tuberculosis* (Mtb), which is accountable for high rates of mortality and morbidity across the globe (166). Dorhoi found that in the blood of patients with active pulmonary TB, miR-223 is abundantly expressed. TB-resistant mice that lack miR-223 expression exhibit high susceptibility to acute lung infection. miR-223 can reverse the mAb depletion of neutrophils by targeting the chemoattractants C-X-C motif chemokine ligand-2 (CXCL2), chemokine C-C motif chemokine ligand 3 (CCL3), and IL-6 to delete chemokine C-X-C motif receptor 2 (CXCR2) (157). Further research revealed that in plasma from patients with pulmonary TB, miR-223 is significantly downregulated. The variance in miR-223 expression levels may be linked to the diversity among the TB strains causing infection (158). As STAT1 has a significant impact in the immune defense against TB infection, the STAT1-related molecule miR-223 has been identified as a potential biomarker in the development of active TB (159). The above studies indicate the important role of miR-223 in the treatment of TB.

Sarcoidosis is a medical condition that is distinguished by the development of granulomas and an exaggerated immune response to unknown agents (167). To identify sarcoidosis biomarkers, Zhou et al. studied microRNA and protein-coding gene expression data of peripheral blood mononuclear cells (PBMCs) in healthy people and patients with noncomplex or complex sarcoidosis. They found that miR-223 may be associated with the severity of sarcoidosis (168). Neli et al. isolated the peripheral blood (PB) and bronchoalveolar (BAL) fluid of sarcoidosis patients for miRNA analysis. Through the analysis of PB’s and BAL’s global transcriptome, they recognize the alteration of miR-223 in TLR-2 and NF-κB apoptosis and proliferation signals. The expression of miR-223 is increased in regulatory T cells (Tregs) from patients with pulmonary sarcoidosis (160). Huppertz et al. recruited 19 healthy volunteers and 19 sarcoid patients, and demonstrated that the mRNA levels of miR-223 are decreased in BALF. And miR-223 KO mice can increase granuloma formation compared to wild-type (161). These data provide strong evidence that miR-223 is an essential part of the network in pulmonary sarcoidosis.

Pulmonary fibrosis is a chronic condition that is defined by the gradual loss of lung function (169). Most cases of fibrosis can be classified as idiopathic (170). Idiopathic pulmonary fibrosis is a lethal form of interstitial pneumonia that is characterized by the gradual scarring of lung tissue (171). Stachowiak et al. included 30 pediatric patients diagnosed with cystic fibrosis and collected their biologic material during pulmonary exacerbation. miRNA profiling showed that miR-223 is significantly altered during pulmonary exacerbation in sputum (162). In the pulmonary fibrosis rat model, Qu et al. found that the model group exhibited a significantly higher pulmonary inflammation score compared to the control group. Furthermore, the expression level of miR-223 decreased with increasing fibrosis (163). However, further research is needed to explore the specific role of miR-223 in pulmonary fibrosis.

## 5. The potential role of miR-223 in clinical treatment

Ever since miRNAs were first discovered in 1993, understanding the function of miRNAs in development and disease has rendered them appealing resources and objectives for innovative diagnosis and prognosis biomarkers, as well as therapeutic strategies.

Due to the multiple functions of miRNAs in disease development, the disorder of one or a group of specific miRNAs may be closely related to human disease progression. Therefore, miRNAs are considered as potential diagnosis and prognosis biomarkers in disease. For example, miR-21, miR-20a, miR-103a, miR-106b, miR-143 and miR-215 et al. are considered as the biomarker in cancer (17), miR-155, miR-27a, miR-21, miR-146a, and miR-223 et al. are proved to play an essential role in regulating ALI/ARDS (172), miR-143, miR133, miR-145, miR-15, miR-126 et al. have been demonstrated to play a part in cardiovascular diseases (173). In recent years, the potential of miR-223 as diagnosis and prognosis biomarkers has also explored (Table 4). Pan et al. found that compared with healthy women, the levels of miR-223 are underrepresented in exosomes from plasma of epithelial ovarian cancer (EOC) patients, this indicates that miR-223 decrease its oncogenic potential in exosomes (174). Another clinical trial reveals that the expression level of miR-223 from the serum of laryngeal squamous cell carcinoma (LSCC) patients is down-regulated (175). In addition to cancer, the application of miR-223 has also been studied in cardiovascular disease. Charlotte et al. first investigates the postprandial responses of miR-223 levels. Their study shows that high-fat meal intake increases miR-223 levels (176). Lidia et al. show that miR-223 macrophage levels in non-smoker men with high cardiovascular risk are increased after beer and decreased after non-alcoholic beer consumption (177). In other diseases, Claudia et al. reveals that bicarbonate hemodialysis (BHD)-derived EV of chronic kidney disease patients had an increased expression of the proatherogenic miR-223 with respect to healthy subjects or mixed online hemodiafiltration (mOL-HDF). The switch from BHD to mOL-HDF significantly reduced systemic inflammation and miR-223 expression in plasma EV, thus improving HUVEC angiogenesis (178). In a double-blind randomized controlled trial of the effects on thrombotic markers and miRNA levels, the author found that compared with aspirin, during prasugrel-therapy of type 2 diabetes mellitus (T2DM), the expression of miR-223 decreased (179). A further study shows that miR-223 from plasma of high dairy and adequate dairy are upregulated. This indicates that miR-223 is related to T2D (180). In addition, an research analyzed miRNAs in cerebrospinal fluid (CSF) from intraventricular hemorrhage (IVH) infants showed that the levels of miR-223 were elevated in CSF after the onset of IVH (181). Hung and his colleges measured the levels of intracellular regulatory miRNAs in PBMCs and monocytes isolated from patients with major depressive disorder (MDD) before and after treatment with antidepressants. They found that the level of miR-223 was increased significantly in those who achieved remission. This suggested that miR-223 may act as biomarkers for remission during the treatment of MDD (182).

**Table 4:** miR-223 in clinical trials.

Target diseases	Intervention measures	Expression of miR-223 as biomarker	References
EOC	N/A	↓ In exosomes from plasma of EOC patients	(174)
LSCC	N/A	↓ In serum of LSCC patients	(175)
Cardiovascular Disease	Theobromine	↑ In apolipoprotein B-depleted serum	(176)
Cardiovascular Disease	Beer or non-alcoholic beer	↑ In macrophage	(177)
Chronic kidney disease	BHD or mOL-HDF	↑ In EV	(178)
T2DM	Aspirin, Clopidogrel, Prasugrel	↓ In serum	(179)
T2D	HD and AD	↑ In serum	(180)
IVH	MetHb, ferrylHb, heme, or TNF-α	↑ In CSF	(181)
MDD	Antidepressants	↑ In PBMC and monocytes	(182)

At present, clinical trials using miRNAs as therapeutic targets mainly focus on tumors and cardiovascular diseases. For cancer diseases, considering the complex interplay between miRNAs and target genes, the targets chosen for ongoing clinical trials typically exhibit confirmed interactions with multiple oncogenes or tumor suppressor genes. Moreover, these miRNAs function as Onco-miRs or Suppressor-miRs across various malignant tumors (6, 183), and several miRNA-targeted therapies are already in clinical development (184–187). For cardiovascular diseases, miRNAs can be used to treat dyslipidemia that increases the risk of cardiovascular disease, such as atherosclerosis (188). It is worth mentioning that, CDR132L, the first miR-132 inhibitor for heart failure has entered clinical trials which marks the entry of miRNA in the treatment of heart disease (189).

At present, strategies that target miR-223 in diseases treatment are mainly in animal models. For example, in the mouse model of KD, the excessive arterial damage could be rescued by administration of miR-223 mimics (190). In addition, *in vivo* and *in vitro*, miR-223 mimics were proved to protect against atherosclerosis by reducing the inflammation and the MEK1/ERK1/2 signaling pathway (191). A recent study also showed that miR-223 mimics could reduce the NLRP3 protein expression level and suppress chronic inflammation in DE (89). For radiation-induced heart disease (RIHD), miR-223 mimics could improve myocardial injury by activating adenosine monophosphate activated protein kinase (AMPK) (192). On the other hand, miR-223 inhibitors could cause hyper-activation of NLRP3 in the J774A.1 murine macrophages infected by subsp. zooepidemicus (SEZ) (193). What is more, miR-223 inhibitors can upregulate the expression of NLRP3 and increase the pyroptosis of fibroblast-like synoviocytes (FLSs) in gout arthritis (GA) rats (194). These examples indicate that in recent years, a considerable number of animal experiments have employed miR-223 mimics and inhibitors. This suggests that the development of miR-223 drugs is not impossible, and the future development and utilization of miR-223 clinical drugs will have significant implications.

As miR-223 plays an important role in pulmonary inflammations, it has great prospects in the clinical treatment of lung inflammations. Therefore, clinical trials of miR-223 as therapeutic targets in pulmonary inflammations are urgently needed.

## 6. Conclusions and future perspectives

In this review, we summarized the role of miR-223 during the inflammatory response in various cell types, as well as the role of miR-223 in some representative pulmonary inflammatory diseases, especially ALI, asthma, COPD and COVID-19. As we mentioned before, during tissue or cellular inflammation, the expression of miR-223, a multifunctional miRNA, is particularly elevated and regulated by several transcription factors. This miRNA is involved in regulating immune cell differentiation, polarization and proliferation, and plays a critical role in mediating cell-tissue, cell–cell, cell-tissue-inflammatory disease interactions. The molecular mechanisms of immune modulation by miRNAs can provide insights into the early diagnosis or treatment of inflammation.

miR-223, which has immunomodulatory effects in some tissues, can regulate immune cell polarization, proliferation and differentiation during pulmonary inflammation. It can also serve as a messenger of inflammation in the immune system. We can provide some insights into the early diagnosis or treatment of pulmonary inflammatory diseases by elucidating the molecular mechanisms of miR-223 in inflammation modulation. By establishing the regulatory network of miR-223, drugs targeted by miR-223 have better efficacy in treating pulmonary inflammatory diseases.

Since December 2019, COVID-19 has emerged as a global pandemic, posing serious public health threat. The symptoms of COVID-19 include fever, cough, myalgia, fatigue, or dyspnea, and elderly people and those with comorbidities are at higher risk of infection, which can lead to severe complications and high mortality rates. In this context, miR-223 has been recognized as a crucial controller of the lung inflammatory response, as it targets a variety of factors such as TLR4, CXCL2, PI3K/AKT, TRAF6, PARP-1, IFN-I, CCL3, STMN1, IKKα, IL-6, IL-1β, IL-18, NLRP3, Caspase-1 and NF-κB. These suggest that miR-223 may play a crucial role in COVID-19 pathogenesis. Since there is no authorized medication available to eliminate the virus, examining miR-223 as a possible target for therapy could provide novel approaches to manage COVID-19.

To sum up, the involvement of miR-223 in lung inflammation has been established, and the information about its targets provides valuable insights that may lead to the development of innovative therapeutic approaches for pulmonary inflammation pathogenesis.

## Author contributions

HF and YZ initiated the project, made suggestions, and revised the article. MS searched the database and wrote the first draft of the manuscript. QL, ZD, SY, JL, MJ, and SH revised and finalized the manuscript. All authors contributed to the article and approved the submitted version.

## Funding

This work was supported by the Scientific Research Translational Foundation of Wenzhou Safety (Emergency) Institute of Tianjin University (TJUWYY2022002 and TJUWYY2022011)，Open Project of the Key Laboratory of Trauma and Orthopedics Research Medicine in Henan Province (HZKFKT20220504), National Natural Science Foundation of China (grant number 32000877), and Open Scientific Research Program of Military Logistics (BLB19J006 and BLB20J009).

## Conflict of interest

The authors declare that the research was conducted in the absence of any commercial or financial relationships that could be construed as a potential conflict of interest.

## Publisher’s note

All claims expressed in this article are solely those of the authors and do not necessarily represent those of their affiliated organizations, or those of the publisher, the editors and the reviewers. Any product that may be evaluated in this article, or claim that may be made by its manufacturer, is not guaranteed or endorsed by the publisher.
